# Cost of managing women presenting with stage IV breast cancer in the United Kingdom

**DOI:** 10.1038/sj.bjc.6601890

**Published:** 2004-06-08

**Authors:** E Remák, L Brazil

**Affiliations:** 1 MEDTAP International, 20 Bloomsbury Square, London WC1A 2NS, UK; 2 Breakthrough Breast Cancer, Institute of Cancer Research and The Royal Marsden Hospital, 237 Fulham Road, London SW3 6JB, UK

**Keywords:** cost of illness, economic burden, incidence, metastatic breast cancer, stage IV breast cancer

## Abstract

This study estimated lifetime cost of treatment for patients in the United Kingdom (UK) presenting with stage IV breast cancer. To determine patterns of treatment and resource use in the absence of direct observational data, a cancer physician panel was surveyed. The survey questionnaire described four predefined treatment phases: active treatment; follow-up after active treatment until disease progression; active supportive care after progression; and end-of-life care. Physicians were asked their major treatment strategies for each phase. Monthly cost and average lifetime cost per patient were calculated. Only five cancer registries in the UK document the proportion of breast cancer patients diagnosed with stage IV disease. Their data was used to estimate the incidence of metastatic breast cancer at presentation throughout the UK. This value, together with lifetime cost per patient and projected survival time, allowed approximation of the overall cost for this population of cancer patients in the UK. Annual incidence of stage IV breast cancer at presentation in the UK is approximately 2100; according to treatment practice in 2002, lifetime cost per patient is £12 500 and total population cost is approximately £26 million. The substantial economic burden associated with patients diagnosed with metastatic breast cancer should be considered when developing strategies for reducing its incidence such as increased awareness, screening and preventative measures.

Female breast cancer caused more than 12 000 deaths in the United Kingdom (UK) in the year 2000 (Cancer Research UK, 2002); in 1998, it was the leading cause of death among women aged 35–54 years (NICE, 2002a). During 1998, there were 32 908 new registrations of female breast cancer in England (Office for National Statistics, 1998).

Breast cancer is staged according to tumour size and degree of local spread, involvement of nearby lymph nodes, and the presence of metastases. A stage IV breast cancer (advanced breast cancer) is one that has metastasised and as such is considered incurable. Common sites of metastasis include the lung, liver, bone, and brain (American Cancer Society, 2003). The variability of its natural history and clinical presentation poses a challenge to any attempt to model its management.

The incidence and prevalence of metastatic breast cancer and its associated morbidity and mortality involve a substantial economic burden. Treatment of stage IV breast cancer is complex, and a number of advances have occurred in the UK in recent years, including the introduction of aromatase inhibitors, taxanes, and trastuzumab. Earlier cost-of-illness studies or studies modelling resource use in metastatic breast cancer (Richards *et al*, 1993; Wolstenholme *et al*, 1998; Dolan *et al*, 1999) may not accurately reflect current treatment patterns. The study described in this paper was designed to investigate the current treatment patterns associated with stage IV breast cancer in the UK and to estimate the economic burden associated with those patients presenting with an advanced stage of the disease.

## METHODS

### Study design

Cost-of-illness studies can be undertaken according to the prevalence approach or the incidence approach. The prevalence approach estimates the total cost of disease in a given year; the incidence approach estimates the lifetime cost of cases first diagnosed in a given year. The incidence approach is more useful for the evaluation of healthcare options in a given time period, as it provides a baseline against which new interventions can be assessed (Drummond, 1992). The study reported here was designed in accordance with the incidence approach to estimate the lifetime cost of treatment for all female patients in the UK with stage IV disease at first diagnosis of breast cancer. Databases analysed included UK breast cancer data from 1994 to 2001. Results were based on estimated incidence of the disease, typical treatment patterns (as determined by members of a cancer physician panel), estimated resource use and the respective standard costs, average length of treatment, and average survival time.

### Incidence

The National Cancer Intelligence Centre at the Office of National Statistics confirmed that no reliable, centrally held information on the separate incidence of each stage of breast cancer in the UK exists. Therefore, in order to estimate the annual incidence of stage IV at presentation breast cancer for this study, we contacted the regional cancer registries (see Appendix). Four English cancer registries (Northern & Yorkshire, East Anglia, Thames (London region), and West Midlands) and the Scottish Cancer registry were able to provide information on breast cancer incidence according to cancer stage. The expected proportion of stage IV cases among all breast cancers at diagnosis was calculated as the average proportion for these five registries, weighted by the total number of registrations at each registry. The total incidence of breast cancer (all stages) in England (Office for National Statistics, 1998), Wales (Office for National Statistics, 1999), and Northern Ireland (Office for National Statistics, 1999) was obtained from the Office for National Statistics; the total incidence in Scotland was provided by the Scottish Cancer Registry (Scottish Cancer Registry, 1998).

Published information on survival time of women with metastatic breast cancer is scarce; survival information used in this study was obtained from the Royal Marsden Hospital database in London. The dataset includes staging information for 2353 female patients seen at Royal Marsden Hospital between 1994 and 2001 who were diagnosed with metastatic breast cancer.

### Treatment patterns and resource use

**Figure 1:**
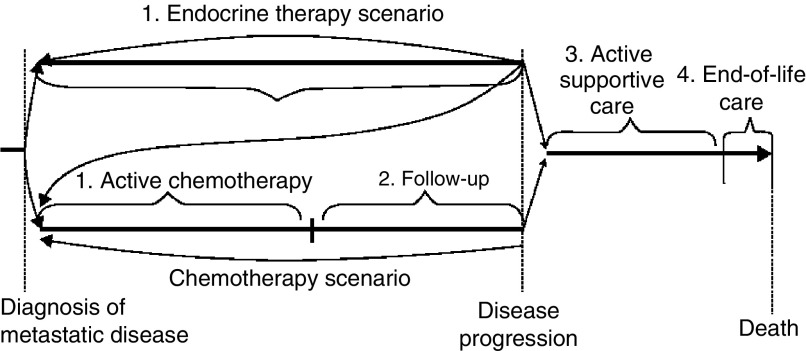
The four treatment phases for stage IV breast cancer: (1) active treatment (endocrine therapy and/or chemotherapy, including multiple cycles); (2) follow-up treatment until disease progression; (3) active supportive care after disease progression; and (4) end-of-life care. (Second-,third-, and further-line active-treatment cycles and subsequent follow-up may also be elected after an instance of disease progression (indicated by backward arrows)).

Information on usual treatment practice was collected from a panel of cancer physicians (see Appendix for more detail). A survey instrument (questionnaire) was designed that distinguished among four predefined treatment phases following the occurrence of metastasis (Figure 1

): (1) active treatment; (2) follow-up after active treatment until disease progression; (3) active supportive care after disease progression; (4) end-of-life care.

It was assumed that following diagnosis of metastatic breast cancer, patients received active treatment in the form of endocrine therapy and/or chemotherapy. Active treatment included first- and second-line treatment and, in some cases, third- and fourth-line treatment. For those who received endocrine therapy, treatment continued until disease progression. Thereafter, second-line hormone therapy, chemotherapy, or supportive care would be offered. For those patients who received chemotherapy, a varying number of treatment cycles would be administered according to the agent used and the treatment response. Patients would be monitored until disease progression. Standard chemotherapeutic agents could have been replaced by trastuzumab, either alone or in combination with a taxane or vinorelbine. Following disease progression, second-line chemotherapy or supportive care would be offered.

Information on the use of radiotherapy and bisphosphonates during the active-treatment phase was also collected in the survey. Several resource elements were listed in each category within each treatment phase (Table 1

**Table 1:** Categories of resource use

**In active-treatment phase**	**In all treatment phases**
• Endocrine therapy	• Palliative radiotherapy
• Chemotherapy	• Medications
○ Monotherapy	• Special interventions
○ Combination therapy	• Investigations, procedures
• Trastuzumab	• Laboratory tests
• Bisphosphonates	• Hospitalisations, hospice visits
• Radiotherapy	• Outpatient visits, other services

).

Participating physicians were asked to indicate the length of each treatment phase and the percentage of patients receiving each type of care. They were also asked to make additions or deletions of care elements to reflect differences in the current management and treatment of stage IV breast cancer patients at their institutions. Where dosage calculations required body surface area or weight, 1.7 m^2^ or 70 kg per patient, respectively, was assumed.

The median was selected *a priori* as the chosen summary measure of resource consumption, with the only exception being the choice of mutually exclusive resource items within treatment groups (ie, where only one drug or procedure, such as a chemotherapeutic regimen, can be chosen at any one time), for which the mean resource consumption was calculated.

Drug costs were based on the British National Formulary (2000), and unit costs for other resources were obtained from the MEDTAP Database of International Unit Costs (2001). Unit costs in the database aim to represent the social opportunity cost of health care resources. Medical staff salaries, overheads as well as equipment costs were included in the calculations. Where necessary, the National Health Service Hospital and Community Inflation Index (PSSRU, 2001) was used to convert these costs to year 2000 prices. Only direct medical costs were included in the cost-of-illness analysis.

### Cost-of-illness calculation

It was assumed that endocrine therapy, chemotherapy, or trastuzumab therapy (with or without chemotherapy) would be administered only sequentially, but that radiotherapy and treatment with bisphosphonates may be given concomitantly with any of the above treatments. Average monthly costs per patient were calculated, using this assumption together with the average treatment lengths indicated by the panel, for a combined active-treatment phase (weighted average of the endocrine therapy and chemotherapy), supportive-care phase, and end-of-life care phase. These monthly costs were then combined with the median survival figure – assuming that all the patients must pass through the supportive-care phase and end-of-life care phase – to obtain the average lifetime cost per patient. This figure was then multiplied by the estimated incidence of stage IV at presentation breast cancer for an estimate of the overall economic burden for this segment of the breast cancer population. As median survival is short, discounting future costs was deemed unnecessary. The robustness of the results was tested in a multivariate sensitivity analysis using Crystal Ball® software (Decisioneering, Inc., Crystal Ball 2000, Standard Edition).

## RESULTS

### Incidence

Reported annual incidence of stage IV at presentation breast cancer from four English registries and the Scottish Cancer Registry is shown in Table 2

**Table 2:** Incidence of *de novo* stage IV breast cancer in four English regions and in Scotland

**Region**	**Year**	**Incidence of breast cancer**	**Incidence of stage IV at diagnosis**	**Proportion diagnosed with stage IV (%)**
Northern & Yorkshire	1998	3964	246	6.21
East Anglia	1998	1834	98	5.34
Thames (London region)	1999	4236	251	5.93
West Midlands	1999	3573	130	3.64
**English registries, combined**				**5.33**
**Scotland**	1998	3523	155	**4.40**
**Overall**				**5.14**

a As reported by four English registries and the Scottish Cancer Registry (see Appendix). Bold values represent the total values.

. These five registries recorded more than 17 000 new cases of breast cancer in 1998 and 1999. The proportion of breast cancer patients presenting with metastatic disease ranged from 3.64% (West Midlands) to 6.21% (Northern & Yorkshire) of all incident cases. Each country's average proportion was used separately to estimate the total incidence of stage IV at presentation cases in England and Scotland, and the overall (combined English and Scottish data; Table 2) average proportion was used to estimate the total incidence of stage IV at presentation cases in Wales and Northern Ireland (Table 3

**Table 3:** Estimated incidence of *de novo* stage IV breast cancer in the UK

**Region**	**Year**	**Incidence of breast cancer**	**Proportion diagnosed with stage IV (%)^a^**	**Incidence of stage IV breast cancer**
England	1998	32 908^b^	5.33	1753
Scotland	1998	3523^c^	4.40	155
Wales	1997	2270^d^	5.14	117
Northern Ireland	1996	873^d^	5.14	45
**UK**		**39 574**		**2070**

a See Table 2.

b Office for National Statistics (2002).

c Scottish Cancer Registry (1998).

d Office for National Statistics (2001).

Bold values represent the total values.

). With these calculations, the total number of newly diagnosed patients in the UK with stage IV breast cancer was estimated to be 2070 (Table 3).

### Treatment patterns and resource use

In total, 21 questionnaires regarding treatment patterns and resource use were mailed to members of the cancer physician panel; 17 (81%) of these were completed and returned. The composition of the panel reflected the specialties of clinicians responsible for treating patients with stage IV breast cancer. Participants included consultants from the fields of Medical Oncology, Clinical Oncology, and cancer research, and they represented a range of hospitals in the north and south of England (see Appendix). Treatment patterns and treatment-associated average monthly costs from survey results are summarised in Table 4

**Table 4:** Treatment patterns and associated costs for stage IV breast cancer in the UK

**Resource category (%)**	**Major resource items within category (%)^a^**	**Average monthly cost per patient within category (£)**	**Percentage of monthly costs per patient (within phase) (%)**
**(a) Active-treatment phase and follow-up phase**			
*Active-treatment phase*			
Endocrine treatment (70%)^b^	Tamoxifen (41%)^c^		
	Aromatase inhibitors (52%)	52.17	3.1%
Chemotherapy (60%)^b^	Combination chemotherapy (45%) Single agent (55%)	403.46	20.7
Trastuzumab (5%)^b^	Alone (68%)^c^ Combination(32%)	1899.34	8.1
Bisphosphonates (50%)^b^		179.95	10.4
Radiotherapy (35%)^b^	Four fractions per annum	19.78	0.8
Medication for supporting:	Chemotherapy	54.09	3.0
	Endocrine therapy	11.91	0.7
Special interventions		29.25	3.4
Scans and laboratory tests		227.63	26.3
Hospitalisations	Endocrine patients (10%)	15.63	0.9
	Chemotherapy patients (20%)	56.25	3.1
Outpatient visits	Specialist visit (100%)	166.79	19.3
	Day case (50%)		
			
*Follow-up phase*			
Medication		9.84	6.3
Scans and laboratory tests		62.91	40.4
Outpatient visits	Specialist visit (100%)	82.90	53.3
	Day case (18%)		
	GP (40%)		
	District nurse (30%)		
			
**(b) Supportive care and end-of-life care**			
*Supportive-care phase*			
Radiotherapy	Average 0.3 fractions month	17.80	2.5
Medication		62.90	9.3
Special interventions		101.66	15.1
Scans and laboratory tests		77.77	11.6
Hospitalisations	42%	157.40	23.4
Outpatient visits	Specialist (90%)	255.20	37.9
	MacMillan nurse (65%)		
	District nurse (50%)		
			
*End-of-life phase*			
Radiotherapy	Average 0.1 fraction	5.93	0.5
Medication		39.86	3.0
Laboratory tests		1.75	0.1
Hospitalisations	20%	395.59	30.0
Hospice stays	70%	498.66	37.9
Outpatient visits	Oncologist (20%)	374.17	28.4
	District nurse (60%)		
	MacMillan nurse (75%)		
	Palliative care physician (50%)		

a Only the major treatment items (those received by >5% of patients) within each resource category are listed.

b Percentage of total patients who received this category of treatment.

c Percentage of those patients receiving this category of treatment who received this specific treatment.

.

According to our results, 70% of patients receive some form of endocrine therapy during the active-treatment phase. The majority of patients receive either tamoxifen (41%) or an aromatase inhibitor (anastrozole, 38%; letrozole, 8%); 60% of patients are treated with chemotherapy, and 5% receive trastuzumab. Anthracyclines (alone or in combination) are the most frequently used chemotherapeutic agents (administered to 55% of chemotherapy patients), whereas approximately 41% of chemotherapy patients receive some form of antimetabolite, usually as part of a combination therapy. Taxanes are administered to 17% of patients, with docetaxel being the first option. Radiotherapy is administered to 35% of patients with metastatic breast cancer. It may be used to treat locally recurrent disease or to palliate bone, brain, or spinal cord lesions. Bisphosphonates have an important role in the treatment of osteolytic lesions, hypercalcaemia, and bone pain associated with skeletal metastases. Half of the population of patients with metastatic breast cancer are treated with bisphosphonates. In terms of resource use and apart from the main therapeutic agents, scans, laboratory tests, and outpatient visits to monitor disease development represent the largest items of care during the active-treatment phase (Table 4).

In the supportive-care phase, the emphasis shifts from active therapies to treatments aimed at alleviating pain and other symptoms. The frequency of blood transfusions and other special interventions increases. The greatest change in terms of resource use can be observed in the increased numbers of hospitalisations, hospice stays, and outpatient visits. During the active-treatment phase, these represent 23.4% of average monthly costs; in the supportive-care phase, they represent approximately 61% of average monthly costs. This trend continues into the end-of-life phase, during which actual medical treatment is almost entirely restricted to administering medication to alleviate symptoms of the disease. Most resource use at this time (96%) is associated with hospitalisations, hospice stays, and outpatient visits. According to survey results, 20% of patients are hospitalised (average length of stay, 7 days), and 70% of patients receive care in a hospice (30%, day visits; 40%, average of five overnight stays) (Table 4).

### Cost-of-illness calculation

Monthly costs for the active-treatment phase and the follow-up phase were combined according to the distribution of treatments indicated in the survey (each with or without follow-up between end of treatment and disease progression) in order to obtain an estimate of average per-patient monthly costs for the entire population of patients. The average monthly costs for each treatment phase are summarised in Table 5

**Table 5:** Monthly costs of stage IV breast cancer per patient

**Treatment phase**	**Estimated length (months)**	**Average monthly cost per patient (£)**
Active^a^	13.53	679
Supportive care	4.00	675
End-of-life	0.47	1316^b^

a Active-treatment and follow-up phases combined.

b Cost per phase.

.

According to Royal Marsden Hospital data, the median survival for patients diagnosed with stage IV breast cancer is 18 months. In the absence of reliable survival estimates for other UK regions, this figure was used to calculate lifetime costs per patient for the entire UK; thus, treatment for metastatic breast cancer was estimated to cost £12 502 (95% confidence interval (CI); £9008–£16 701) over the lifetime of each patient. Translating this individual cost to the population level, it was calculated that treating all patients in England that present with stageIV breast cancer in 1 year costs approximately £22 million, and treating all patients throughout the UK costs approximately £26 million (Table 6

**Table 6:** Population-level cost of stage IV breast cancer in the UK

**Region**	**Annual incidence of stage IV breast cancer**	**Annual cost per population (£)**
England	1753	21 920 115
Scotland	155	1 937 899
Wales	117	1 457 883
Northern Ireland	45	560 675
**UK total**	**2070**	**25 876 572** (95% CI: 13 316 695–38 936 650)

Bold values represent the total values.

).

In the sensitivity analysis, the variable for incidence of stage IV at presentation breast cancer was allowed to follow a normal distribution, with the 5% tile being 1440 cases per annum (assuming that all of the UK experiences the lower incidence rate reported by the West Midlands registry), and the 95% tile being 2456 cases (assuming that all of the UK experiences the higher incidence rate reported by the Northern & Yorkshire registry) (Table 2). The length of each predefined treatment phase and its associated costs were allowed to vary according to individual responses from the 17 clinicians participating in the resource use survey. The median survival was allowed to follow a normal distribution, with a mean of 18 months and a standard deviation of 3 months. The resulting 95% CI for annual costs is reported in Table 6.

## DISCUSSION

The present study estimated the direct cost of lifetime treatment of all patients in the UK presenting with stage IV breast cancer within a given year at approximately £26 million. This undoubtedly underestimates the total economic burden that stage IV breast cancer places on the health-care system for two reasons. First, cancer registries are not always able to provide complete information: registry databases do not include all patients with cancer, nor do they include the stage at diagnosis for every patient registered. For example, the stage at diagnosis was unknown in 44% of the new breast cancer registrations made in 1999 by the Thames Cancer Registry for residents of the London region. Therefore, a certain number of stage IV patients might not have been registered at all or might have been registered but not classified as having stage IV disease. Second, about 50% of those patients diagnosed (and registered) with an earlier disease stage will later develop metastases (Blum, 2001). However, disease progression is not documented by the cancer registries, therefore, the estimates are necessarily limited to the population of patients with stage IV disease at the time of breast cancer diagnosis. This figure is still important economically and is useful to know for helping to develop increased awareness, better screening/detection and preventative measures especially in high-risk groups.

As an exploratory analysis, we also estimated the overall number of patients with stage IV breast cancer. Using incidence and survival information from the Royal Marsden Hospital database, assuming that 50% of patients will develop stage IV disease 18 months (the median survival for those diagnosed with stage IV disease) before death, and assuming no changes in incidence of breast cancer, the prevalence of stage IV breast cancer was estimated at about 29 500 once steady state is reached. The crude estimate of annual cost of treatment for metastatic breast cancer is then over £245 million.

Prospectively observed treatment patterns for patients with stage IV breast cancer would be preferable to estimates derived from surveying a cancer physician panel. Although there is reasonable geographical spread in selected physicians, the selection was random. The panel included physicians with breast cancer as their main interest, but it was a general panel reflecting the multidisciplinary practice for breast cancer. Physicians were asked to describe treatment practices in their hospital, not their own views, and there was no incentive to answer in a particular way or in favour of a particular treatment, but there is potential for bias as only those with enough time to complete the lengthy questionnaire participated. Nonetheless, in the absence of observed treatment patterns, the estimates presented here provide information on the major resource items used and the associated treatment costs for patients with stage IV breast cancer.

We identified two other studies that calculated lifetime costs of stage IV breast cancer in the UK (Richards *et al*, 1993; Wolstenholme *et al*, 1998). Richards *et al* analysed the medical records of 50 patients with advanced breast cancer at Guy's Hospital Oncology Unit. The median duration of advanced disease was 17 months and the mean cost of treatment per patients was calculated to be £7620 (range, £317–£27 860). Wolstenholme *et al* took a random sample of the case notes of 137 patients diagnosed with breast cancer in 1991 from the Trent Cancer Registry. Only six of these patients were diagnosed with stage IV disease, and the range of the cost estimate was very wide. The mean total cost of diagnosis and treatment for stage IV disease over 4 years – £6591 in 1991 prices – was significantly higher than that for any of the earlier stages. Dolan *et al* (1999) used this information to calculate secondary care costs for breast cancer in the UK. They estimated that there were 1456 women with stage IV disease in 1995–1996, costing the National Health Service a total of almost £13 million. Inflating these costs to prices in the year 2000 using the National Health Service Hospital and Community Inflation Index (PSSRU, 2001) brings the cost reported by Richards *et al* to £9555 (range, £397–£34 935) and the cost reported by Wolstenholme *et al* to £9002 (range, £1276–£11 904). These figures are comparable to our results. A number of factors can explain the slight difference: for example, the above estimates did not include the costs of general practitioner visits or palliative care, both of which we observed to play an important role, especially in the last two treatment phases; furthermore, a small samples may not be representative of a population with considerable survival and treatment variability.

Although metastatic breast cancer is considered incurable, survival may range from a few months to several years. Analysis of a series of patients with recurrent disease indicates that survival is related to the sites of metastasis. Visceral recurrences were associated with shorter survival; patients with bone and soft tissue metastases had a better prognosis (Leone *et al*, 1988; Perez *et al*, 1990). A multivariate analysis of prognostic factors for survival in 439 women with recurrent breast cancer showed that the following factors were associated with shorter survival from the time of the first recurrence: site of recurrence; four or more axillary lymph nodes involved at initial diagnosis; negative oestrogen receptor status; and disease-free interval (time from diagnosis of primary tumour to recurrence) of less than 60 months (Insa *et al*, 1999). The dominant site of metastasis was also an important determinant for response to treatment and survival in a Cox proportional hazard model analysis of 371 patients (Nomura, 1998). Having a single bone lesion, rather than multiple bone lesions or additional visceral involvement, seems to be associated with the best outlook for survival. Durr *et al* (2002) followed 70 patients with breast cancer who were surgically treated for bone metastasis. For the entire group (including those with one or multiple bone lesions and 32 patients with additional visceral involvement), the 5-year survival rate was 13%, whereas patients with solitary bone lesions had a 39% chance of 5-year survival.

Treatment of metastatic breast cancer is growing increasingly complex with the recent introduction of several new treatment options. Since these novel therapies come at a relatively high price, it is not surprising that they feature prominently on the agenda of the National Institute for Clinical Excellence (NICE). In 2001, NICE recommended the use of the taxanes, docetaxel and paclitaxel, as an option for the treatment of patients with advanced breast cancer for whom cytotoxic chemotherapy (including an anthracycline) has failed or is judged inappropriate (NICE, 2001). In 2002, NICE also recommended that trastuzumab be available for women with advanced breast cancer and high levels of HER2. The recommendation is to administer trastuzumab either in combination with paclitaxel for women who have not had chemotherapy and for whom anthracycline treatment is not appropriate, or as monotherapy for women who have had at least two chemotherapy treatments for metastatic breast cancer. The previous therapy must have included at least an anthracycline and a taxane, if appropriate; for patients sensitive to oestrogen, it should also have included hormonal therapy (NICE, 2002a). For combination therapy, trastuzumab is currently licensed for use only with paclitaxel, but our survey showed off-label use with docetaxel and with vinorelbine. However, a recent comprehensive audit of availability of breast cancer drug shows huge variations in access across the UK (CancerBACUP, 2003). Nationally, only around 33% of women who may benefit from trastuzumab are receiving it. This finding may explain why trastuzumab treatment was indicated for only 5% of patients in our treatment survey. This figure is likely to rise as changes in treatment patterns lead to more patients receiving relatively more expensive treatments. Greater use of trastuzumab and the taxanes will increase the overall cost of treatment for metastatic breast cancer in the UK. According to data provided in the industry submission during the NICE appraisal procedure, trastuzumab is likely to add £15.8 million to the health-care budget. Assessments of other therapies for advanced cancer are in progress: a technology guidance appraisal of vinorelbine and capecitabine use for advanced breast cancer was recently published by NICE (NICE, 2002b; 2003).

For women with oestrogen-receptor-positive breast cancer, tamoxifen has long been the treatment of choice. A relatively new strategy for blocking oestrogen action in postmenopausal women is aromatase inhibition (i.e., blocking oestrogen synthesis). Aromatase inhibitors are licensed for second-line treatment for those not responding to, or otherwise inappropriate for, tamoxifen therapy. However, clinical evidence is emerging that the aromatase inhibitors letrozole and anastrozole may be similar or superior to tamoxifen for first-line treatment for metastatic breast cancer (Bonneterre *et al*, 2000; Nabholtz *et al*, 2000; Mouridsen *et al*, 2001). Notably, the monthly cost of anastrozole or letrozole is more than 10 times that of tamoxifen. Increased use of these agents raise the costs of treatment for metastatic breast cancer considerably, and costs are likely to increase further as their role in therapy continues to grow.

## CONCLUSION

Information about the incidence of advanced breast cancer is scarce; additionally, considerable variability exists in the treatment patterns and survival time for patients with stage IV breast cancer, depending on factors such as the site and number of metastases and the individual physician's approach to therapy. The present study provides estimates of the incidence of newly diagnosed metastatic breast cancer in the UK and of the cost of treatment of these cases. Stage IV breast cancer imposes a substantial economic burden in the UK, and this burden should be taken into consideration when developing strategies such as awareness programmes, screening, and prevention to reduce the incidence of this disease.

## Appendix

In order to inform on treatment patterns and costs associated with metastatic breast cancer, a panel of cancer clinicians was consulted with the help of a survey instrument. Physicians were randomly selected from the Medical Directory based on their listed specialty. Year 4–5 Specialist Registrars (i.e. not Junior Registrars) or Consultants were invited, who see 50–100 breast cancer cases per month. A maximum of three physicians were accepted from each hospital. A total of 56 clinicians were invited to participate, of whom 35 (63%) declined due to other work-related responsibilities. 21 questionnaires were posted.

An initial survey instrument was developed that included the core components of care associated with each treatment phase. Professor Michael Lind, Consultant Oncologist at The Princess Royal Hospital, Sutton acted as a consultant on both the layout and contents of the questionnaire, and the final survey instrument was developed based on his comments. The panel was asked to think of a group of ‘typical’ patients and provide a description of the treatment these patients would receive in their institution. The medical history and health status of a typical patient was described before each phase. The aim was to gather information on usual clinical practice as opposed to best practice, as usual practice is more informative for determining actual cost of treatment.

Physicians were paid for the time only, receiving £360 for approximately 3 h of work. No additional remuneration was offered. Physicians were not asked to comment on specific drugs or procedures.

### MEMBERS OF THE CANCER PHYSICIAN PANEL

Dr A Abdussalam (Airedale General Hospital, Keighley); Dr T Ahmed (Institute for Cancer Research, London); Dr J Brock (Clattenbridge Centre for Oncology NHS Trust, Merseyside); Dr R Burcombe (Mount Vernon Hospital, Middlesex); Dr DM Carnell (Mount Vernon Hospital, Middlesex); Dr A Chalmers (Gray Cancer Institute, Middlesex); Dr S Cleator (Institute for Cancer Research, London); Dr M Clemons (Christie Hospital, Manchester); Dr D Dodwell (Cookridge Hospital, Leeds); Dr D Fairlamb (New Cross Hospital, Wolverhampton); Dr I Fernando (University Hospital Birmingham NHS Trust, Birmingham); Dr J Larkin (Royal Marsden Hospital, Surrey); Dr Nihal Shah (Royal Marsden Hospital, London); Dr A Sibtain (Mount Vernon Hospital, Middlesex); Dr JA Stewart (Northampton General Hospital, Northampton).

### CANCER REGISTRIES

East Anglian Cancer Registry; Merseyside and Cheshire Cancer Registry; North Western Cancer Registry; Northern and Yorkshire Cancer Registry; Oxford Cancer Intelligence Unit; South and West Cancer Intelligence Service; Thames Cancer Registry; Trent Cancer Registry; West Midlands Cancer Intelligence Unit; Welsh Cancer Intelligence and Surveillance Unit; Scottish Cancer Registry; Northern Ireland Cancer Registry.
